# Human recombinant soluble ACE2 (hrsACE2) shows promise for treating severe COVID­19

**DOI:** 10.1038/s41392-020-00374-6

**Published:** 2020-11-03

**Authors:** Tarek Mohamed Abd El-Aziz, Ahmed Al-Sabi, James D. Stockand

**Affiliations:** 1 Department of Cellular and Integrative Physiology, University of Texas Health Science Center at San Antonio, San Antonio, TX 78229-3900 USA; 2 Zoology Department, Faculty of Science, Minia University, El-Minia, 61519 Egypt; 3 College of Engineering and Technology, American University of the Middle East, Kuwait, Kuwait

**Keywords:** Infection, Infectious diseases, Diagnostics

A recent study by Zoufaly et al. published in *The Lancet Respiratory Medicine* describes encouraging data from the first severe COVID-19 patient successfully treated with human recombinant soluble angiotensin-converting enzyme-2 (hrsACE2).^1^ The published data document upon treatment of an adaptive immune response, the disappearance of the virus swiftly from the serum, the nasal cavity and lungs, and a reduction of inflammatory cytokine levels that are critical for COVID-19 pathology. Notably, the use of hrsACE2 did not impede the generation of neutralizing antibodies, leading to a significant clinical improvement of the treated patient.

A pandemic spread of the severe acute respiratory syndrome coronavirus-2 (SARS-CoV-2) is responsible for more than one million deaths due to COVID-19. Therefore, important insights into the viral pathophysiology may facilitate the search for an effective vaccine and treatment option. In addition to finding viral replication inhibitors, another strategy is to block the cellular target of the virus, angiotensin-converting enzyme-2 (ACE2).^2^

**Fig. 1:**
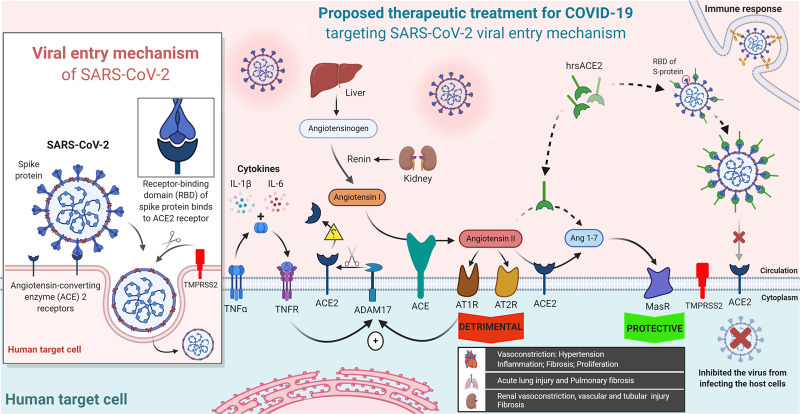
Schematic diagram of the renin-angiotensin system and the proposed therapeutic treatment for COVID-19 targeting SARS-CoV-2 viral entry mechanism. (Left) The receptor-binding domain (RBD) of the spike protein from SARS-CoV-2 binds to ACE2, allowing host cell entry and infection. TMPRSS2 indicates transmembrane protease serine 2. (Middle) The physiological role of ACE2 in renin-angiotensin system and its protective effect on organs. The protease renin, an enzyme produced by the juxtaglomerular cells of the kidney, cleaves angiotensinogen (a precursor of angiotensin produced by the liver) to generate angiotensin I. ACE plays an important role in converting angiotensin I into angiotensin II. Angiotensin II may exert some biological functions through angiotensin II receptor type 1 and 2 receptors (AT1R and AT2R), leading to potent vasoconstriction in several organs. Activation of AT1R increases the transmembrane proteinase (ADAM17) activity. Furthermore, tumor necrosis factor-α (TNF-α) activation of its tumor necrosis factor receptor (TNFR) represents another pathway increasing ADAM17 activity. TNF-α along with cytokines released due to SARS-CoV-2 infection can lead to a cytokine storm. ADAM17 cleaves the extracellular juxta-membrane region of ACE2, whether such ACE2 cleavage contributes to SARS pathogenesis is not known yet. ACE2 hydrolyzes Angiotensin II to the vasodilator Angiotensin 1–7, which binds the Mas receptor and plays a protective role in several organs. The balance between ACE/Ang II/AT1R and ACE2/Ang 1–7/MasR is vital for maintaining normal health. (Right) According to recent studies, increasing of hrsACE2 at tissue sites can effectively compete with the endogenous ACE2 and limits SARS-CoV-2 entrance into the host cells and decreases angiotensin II levels.^1,4^ Significantly, hrsACE2 injection did not reduce the generation of anti-SARS-CoV-2 IgA and IgG antibodies

ACE2 is a crucial receptor target of SARS-CoV-2, which plays a vital role in the pathogenesis of COVID-19, as it enables viral entry into target cells (Fig. 1). The binding affinity between ACE2 and the receptor-binding domain (RBD) of the SARS-CoV-2 spike glycoprotein is 10- to 20-fold higher compared to that with the RBD of SARS-CoV, which likely underpins the higher pathogenesis of SARS-CoV-2 infections. ACE2 is a transmembrane protein typically known for its carboxypeptidase activity and its physiological role in the renin-angiotensin system. ACE2 hydrolyzes angiotensin II to its metabolite, angiotensin 1–7 and angiotensin I to angiotensin 1–9 to protect diverse tissues from injury (Fig. 1).^3^ ACE2 is expressed in several human organs at varying levels. It is highly expressed in the lungs (on the surface of type II alveolar epithelial cells), heart (on myocardial cells, coronary vascular endothelial cells, and vascular smooth muscle), kidney (on proximal tubule cells), and small intestine (on the enterocytes).

While membrane-bound ACE2 may mediate cell entry of SARS-CoV-2, a genetically modified soluble form of ACE2, called hrsACE2, may decrease cell entry of SARS-CoV-2 competing for membrane-bound ACE2. APN001 is a hrsACE2 designed by Apeiron Biologics to imitate the human enzyme ACE2. As such, it may decrease cell entry of SARS-CoV-2 to minimize lung injury, and multiple organ dysfunction (Fig. 1). Experimental support for this theoretical idea has come from in vitro studies showing that hrsACE2 reduces viral growth of SARS-CoV-2 by a factor of 1000–5000 in cell-culture, engineered human blood vessels and kidney organoids.^4^ To date, hrsACE2 has been documented to be safe and tolerable in 89 healthy volunteers in phase-I studies and patients with acute respiratory distress syndrome in phase-II clinical studies. APN01 as a promising therapeutic against COVID-19 has appealing potential and sound underlying scientific rationale.

Here, Zoufaly and colleagues described a case of a 45-year-old woman that was hospitalized with a 7-day history of cough, fatigue, muscle aches, fever, and severe shortness of breath, in addition to 4-day history of nausea and diarrhea. She was diagnosed as having COVID-19 through a reverse transcriptase-polymerase chain reaction (RT-PCR) from a nasopharyngeal swab. After this diagnosis, she was treated with hydroxychloroquine and the anticoagulant, nadroparin. This treatment was ineffective providing no clinical change in the patient’s condition with the chest x-rays demonstrating increasing bilateral, multifocal, and peripheral ground-glass opacities. Nine days after the onset of symptoms, the patient received hrsACE2 twice daily for 5 min by intravenous infusion. Administration of hrsACE2 was continued as scheduled for 7-days and was well tolerated with no clear drug-related side effects. A marked reduction in serum angiotensin II levels with concomitant increases in angiotensin 1–7, angiotensin 1–9, and their metabolite angiotensin 1–5 was observed after the first dose of hrsACE2. These changes were sustained through the observation period. Significant ACE2 activity was observed 7-days after administration of the last dose of hrsACE2. In addition, marked decreases in the concentrations of critical cytokines implicated in COVID-19 pathology to include interleukin IL-6, chemokine IL-8, as well as the soluble receptor for advanced glycation end product, the inflammation marker ferritin, tumor necrosis factor α, surfactant protein-D, C-reactive protein, and angiopoietin 2 were observed. The copy number of SAR-CoV-2 decreased dramatically from 32,000 copies per mL 2 days before administration of hrsACE2 to 2,500 and 270 copies per mL after the first and second day of hrsACE2 treatment, respectively, with rapid clearance from the patient’s plasma during daily testing until the end of the observation period. Furthermore, hrsACE2 injection did not reduce the generation of anti-SARS-CoV-2 IgA and IgG antibodies. Angiotensin II levels returned to pre-treatment levels within 48 h after cessation of hrsACE2, matching previous data on its half-life in humans.^5^ On day 57, the patient was discharged from the hospital after significant clinical improvement.

While promising, we must be mindful that this represents a single observation. Nonetheless, the results, in this instance, clearly demonstrate that SARS-CoV-2 disappeared rapidly from the serum and gradually from the nasal cavity and lungs following hrsACE2 treatment. Whether this marked reduction in viral load reflects the effect of hrsACE2 or the natural course of the disease in this patient is unknown. Most importantly, the use of hrsACE2 did not reduce the generation of neutralizing antibodies. Similar data were observed in a second patient with severe COVID-19 symptoms that received two doses of hrsACE2 for 1 day. Rapid reduction of viral load in the serum along with the generation of antiviral antibodies were observed in this second patient. In addition to reducing the viral load in the respiratory system, hrsACE2 also may have a significant role in slowing or even preventing the systemic spread of the virus from the lungs to other organs, to include possibly reducing attacks by SARS-CoV-2 on the lining of blood vessels.^4^ Although no-clear hrsACE2-related side effects were reported but reduced angiotensin II formation, due to the overexpression of ACE2, may lead to hypotension and acute kidney injury. As with any emerging drug, further research is required to reveal the full potential of hrsACE2 as a sound therapeutic tool, but initial clinical observations are promising.
